# Patients with subclinical hypothyroidism before 20 weeks of pregnancy have a higher risk of miscarriage: A systematic review and meta-analysis

**DOI:** 10.1371/journal.pone.0175708

**Published:** 2017-04-17

**Authors:** Yibing Zhang, Haoyu Wang, Xifeng Pan, Weiping Teng, Zhongyan Shan

**Affiliations:** The Endocrine Institute and The Liaoning Provincial Key Laboratory of Endocrine Diseases, Department of Endocrinology and Metabolism, The First Hospital of China Medical University, Shenyang, China; University of Rochester, UNITED STATES

## Abstract

**Objective:**

To evaluate the relationship between subclinical hypothyroidism (SCH) and the risk of miscarriage before 20 weeks of pregnancy.

**Methods:**

Literature databases were searched, including the PubMed, Web of Science, Embase and Cochrane databases, from January 1, 1980, to December 31, 2015. The following search terms were used: subclinical hypothyroidism, hypothyroidism, thyroid dysfunction, thyroid hypofunction, subclinical thyroid disease, thyroid dysfunction, pregnancy loss, abortion and miscarriage. Studies comparing the prevalence of miscarriage in pregnant women with SCH with those who were euthyroid were selected. From the studies matched, the relative risk (RR) and corresponding 95% confidence interval (95% CI) were calculated to yield outcomes. All the statistical analyses were performed using Review Manager (Revman) Version 5.3 and Stata Version 12.0 software. The publication bias of the studies was assessed by forest plot and Begg’s test, while the stability of the results was evaluated by sensitivity analysis.

**Results:**

Nine articles satisfying the inclusion criteria were analysed. Compared to euthyroid pregnant women, patients with non-treated SCH had a higher prevalence of miscarriage (*RR* = 1.90, 95% *CI*1.59–2.27, *P*<0.01). Additionally, SCH patients in the international diagnostic criteria group were more likely to suffer miscarriages than those in the ATA diagnostic criteria group (χ^2^ = 11.493, *P*<0.01). Moreover, there was no difference between patients with treated SCH and euthyroid women (*RR* = 1.14, 95% *CI*0.82–1.58, *P* = 0.43). Compared to isolated SCH women, the miscarriage risk of SCH patients with thyroid autoimmunity (TAI) was obviously higher (*RR* = 2.47, 95% *CI*1.77–3.45, *P*<0.01), and isolated SCH patients also had a higher prevalence of miscarriages than euthyroid women (*RR* = 1.45, 95% *CI*1.07–1.96, *P* = 0.02).A heterogeneity test, forest plot and Begg’s test suggested that there was no significant heterogeneity or publication bias among the included articles, while the result of sensitivity analysis showed that our study exhibited high stability.

**Conclusion:**

SCH is a risk factor for miscarriage in women before 20 weeks of pregnancy, and early treatments can reduce the miscarriage rate. Regardless of the diagnostic criteria used, the miscarriage rate increased as long as a pregnant woman was confirmed to have SCH. The results show that the omission diagnostic rate may increase when the ATA diagnostic criteria are used. In addition, SCH patients with TAI have a higher prevalence of miscarriage, while isolated SCH patients also have a higher miscarriage rate than euthyroid women. Thus, we recommend early treatments to avoid adverse pregnancy outcomes and complications.

## Introduction

Thyroid dysfunction is prevalent in pregnant women, with a morbidity of 2–3%; it is always caused by chronic autoimmune thyroiditis. Moreover, 5–15% women of reproductive age are diagnosed with thyroid autoimmunity, leading to high risk of adverse pregnancy outcomes[1]. Hypothyroidism is the most common type of thyroid dysfunction, and subclinical hypothyroidism (SCH) has a higher prevalence than overt clinical hypothyroidism (OH). The diagnosis of SCH usually relies on laboratory tests due to the lack of significant clinical features of subclinical diseases, and the non-specific performance in pregnancy patients may be associated with lifestyle changes or manifestations brought out by pregnancy itself[2]. Compared to OH, the incidence of complications related to SCH is lower. However, the prevalence of adverse outcomes, including spontaneous miscarriage, placental abruption, preterm birth, foetal distress and preeclampsia, has increased in recent studies[3]. Whether the miscarriage rate in pregnant women with SCH will increase is still controversial. A large-scale cohort study including 10990 pregnant women found that there was no correlation between SCH and miscarriage or other adverse outcomes[4]. Su et al. conducted a follow-up study among 1017 Chinese pregnant women and came to a similar conclusion[5]. However, several large-scale studies carried out by Liu et al.[6] and Negro et al.[7] revealed that SCH significantly increased the risk of miscarriage in pregnant women. Additionally, the risk of miscarriage increased with the elevation of the serum TSH level, which is consistent with Benhadi et al[8]. Therefore, it is necessary to perform a meta-analysis to evaluate the relationship between SCH and the miscarriage rate in women before 20 weeks of pregnancy by combining the data of all relevant studies.

## Materials and methods

### 1. Literature search strategy

Literature databases, including the PubMed, Web of Science, Embase and Cochrane databases, were searched for relevant studies published from January 1, 1980, to December 31, 2015. **The search strategy we used in this study is ‘(subclinical hypothyroidism OR hypothyroidism OR thyroid dysfunction OR thyroid hypofunction OR subclinical thyroid disease OR thyroid disfunction) AND (pregnancy loss OR abortion OR miscarriage)’**. Additionally, the retrospective method was used to find other eligible studies.

### 2. Inclusion criteria

Cohort studies conducted among women with SCH or normal thyroid function before 20 weeks of pregnancy were included. ‘Abortion’ or ‘miscarriage’ was clearly defined as the event outcome and the primary data from cases and controls could be extracted to calculate the relative risk (RR), 95% confidence intervals (95% CI) and P values.

### 3. Data extraction

The following information was carefully extracted from each study independently by two authors: name of first author, publication year, country of population, diagnostic criteria of SCH, pregnancy phase and numbers of SCH cases and normal controls. Disagreements between two authors were resolved by discussion among the research team.

### 4. Quality assessment of the included studies

The Newcastle-Ottawa Scale (NOS) scoring system was used by two authors to assess the quality of the included literature. The scoring system is a type of bias risk assessment tool recommended by the Cochrane Collaboration and applied in the evaluation of case-control and cohort studies. Three major scoring items, including selectivity, comparability and outcomes, were assessed. As this meta-analysis focused on the relationship between SCH and miscarriage, we defined ‘no history of disease’ as ‘no history of miscarriages or abortions’. The NOS scores ranged from zero to nine stars. All the included studies were assessed by two authors, and disagreements were resolved by discussion with the research team. Additionally, a funnel plot was created to assess the potential publication bias.

### 5. Statistical analysis

Review manager 5.3 software was used for meta-analysis, and the association of SCH in pregnancy and miscarriage was evaluated by odds ratio (OR) and 95% confidence interval (CI). Heterogeneity between studies was analysed by the Cochrane Q test, and P<0.1 suggested statistical significance. At the same time, quantitative analysis of heterogeneity was performed by calculating I^2^. I^2^ values exceeding 25%, 50% and 75% were defined as low, moderate and high heterogeneity, respectively. The random effects model was used when I^2^> 50% and the fixed effects model was used when I^2^<50%. Stata 12.0 software was used to conduct Begg's test and sensitivity analysis in order to evaluate the publication bias in the included studies. This meta-analysis follows the PRISMA 2009 (S1 File).

## Results

### 1. Characteristics of included studies

**Fig 1:**
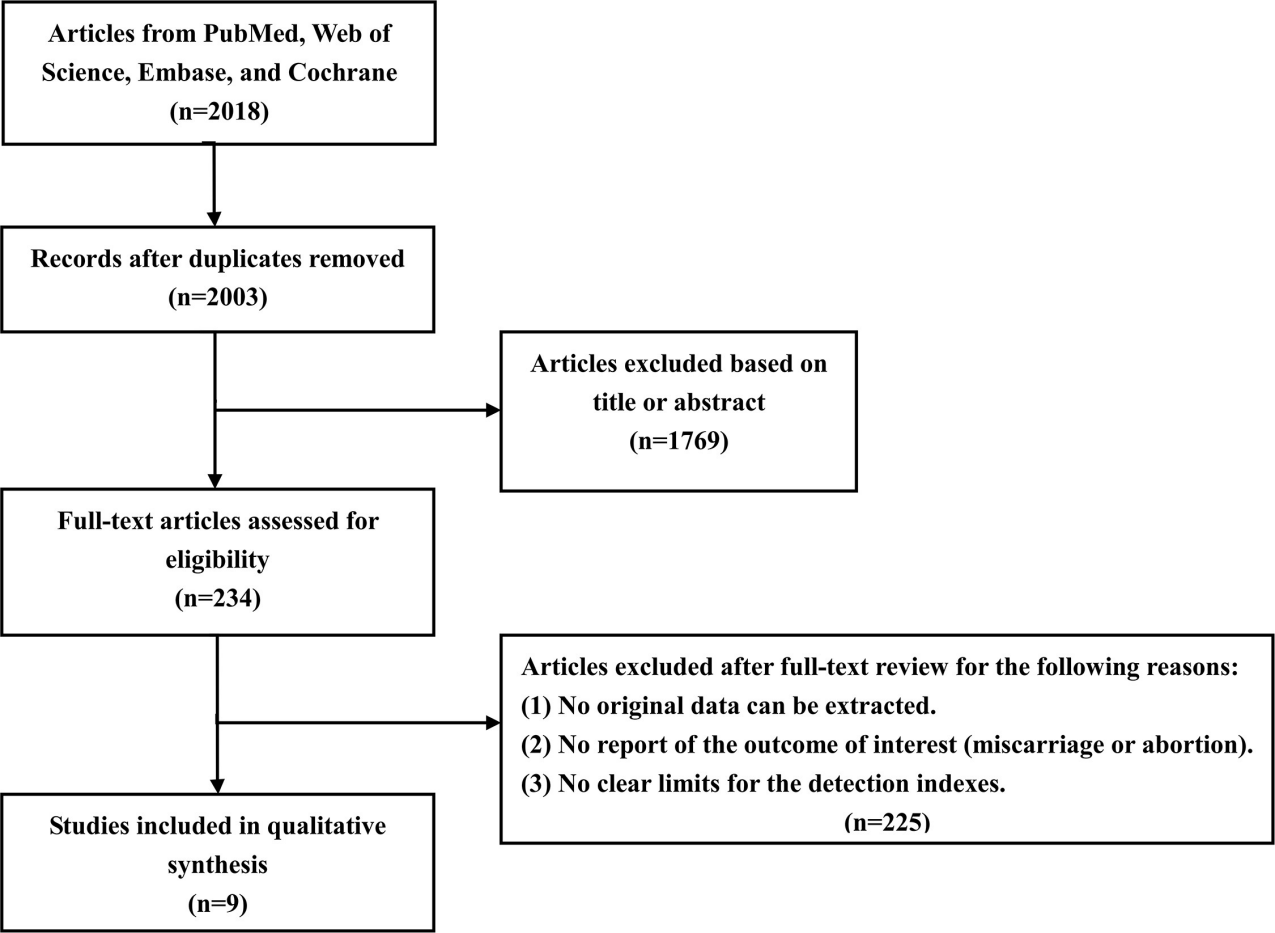
Flow diagram of the literature search.

A total of 2018 articles were retrieved from the PubMed, Web of Science, Embase and Cochrane databases by keyword search A total of 1769 unrelated articles were excluded after reading the titles and abstracts. After full-text review of 249 articles and the removal of 24 duplicate documents, eventually 9 cohort studies met the inclusion criteria and were included in this systematic review. The detailed selection procedures are shown in Fig 1. The eligible studies were published between 2008 and 2015 and proved to be high quality according to the NOS scoring system. The characteristics of each study are shown in Table 1.

**Table 1:** General characteristics of the studies included in this meta-analysis.

Authors	Year	Country	Diagnostic criteria for SCH	Pregnancy Phase	SCH without intervention(n/N)^a^	SCH with treatments(n/N)^a^	SCH+TAI (n/N)^a^	Isolated SCH (n/N)^a^	Euthyroid (n/N)^a^
Cleary-Goldman et al.[4]	2008	U.S.	TSH>97.5th percentile (>4.29 mIU/L), FT4 2.5–97.5th percentile	10–13	1/240	-	-	-	60/10021
Su et al.[5]	2011	China	TSH>95th percentile(TSH>3.6 mIU/L), FT4 5-95th percentile	<20	2/41	-	-	-	11/845
Wang et al.[9]	2012	China	TSH>2.5 mIU/L, FT4 within the normal range	<12	26/168	2/28	-	-	48/542
Aguayo et al.[10]	2013	Spain	2.5 mIU/L<TSH<10.0 mIU/L, FT4 within the normal range	Early pregnancy	45/288	-	-	-	157/1761
Ajmani et al.[11]	2013	India	TSH>3.0 mIU/L, FT4 within the normal range	13–26	-	2/36	-	-	8/347
He et al.[12]	2014	China	TSH>2.5 mIU/L, FT4 within the normal range	<12	32/635	-	30/318	12/317	8/398
Liu et al.[6]	2014	China	2.5 mIU/L<TSH<10.0 mIU/L, FT4 within the normal range	4–8	54/959	-	23/204	31/755	43/1961
Oztas et al.[13]	2015	Turkey	TSH>2.5 mIU/L, FT4 within the normal range	Early pregnancy	-	3/27	-	-	3/56
Yang et al.[14]	2015	China	TSH>97.5th percentile (5.22–10 mIU/L), FT4 2.5–97.5th percentile	<12	46/806	49/1236	23/201	23/605	72/2000

^a^n, the number of patients with SCH in each group; N, the total number of subjects in each group.

### 2. Meta-analysis results

#### (1) SCH without interventions and miscarriage

Seven studies reported relevant data on the association between miscarriage and SCH without intervention. Of 3137 SCH patients, 206 women suffered miscarriage, and of 17528 euthyroid women, 399 women suffered miscarriage. The results of the meta-analysis showed that the prevalence of miscarriage in SCH patients was significantly higher (RR = 1.90, 95% CI1.59–2.27, P<0.01). The result of heterogeneity tests were Chi^2^ = 5.94, P = 0.43, I^2^ = 0%, indicating that there was no obvious heterogeneity among the included studies.

**Fig 2:**
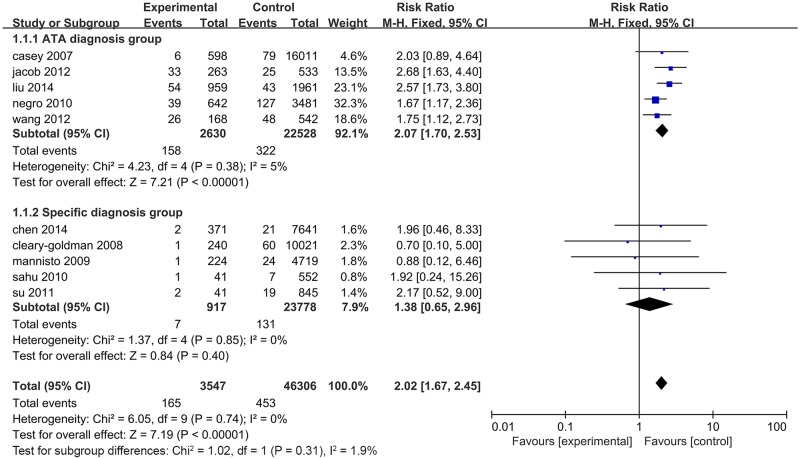
Forest plots for the prevalence of miscarriage risk in untreated SCH patients diagnosed by ATA criteria and specific criteria compared with controls.

Due to the difference in the TSH level used to define SCH, we classified these 7 studies into two subgroups according to the diagnostic criteria. Studies using the American Thyroid Association (ATA) recommended criteria as TSH>2.5 mIU/L in early pregnancy were considered as the ATA diagnosis group and those using TSH greater than the 95th percentile or 97.5th range in healthy women to define SCH were the specific diagnosis group. The association of SCH and miscarriage was analysed in each group, and the prevalence of miscarriage in patients from both groups was clearly higher than euthyroid women (RR = 2.04, 95% CI 1.66–2.51, P<0.01 vs. RR = 1.58, 95% CI 1.12–2.32, P<0.01). Additionally, there was no heterogeneity observed between subgroups (Fig 2). To further assess the different risks of miscarriage induced by two diagnostic criteria, SPSS 22.0 software was used to evaluate the association of miscarriage rates in two groups by the Chi-square test. The incidence of miscarriage in the ATA diagnosis group (7.6%) was significantly higher than that in the specific diagnosis group (4.5%) (χ^2^ = 11.493, *P*<0.01).

Because the included studies were geographically diverse, another subgroup analysis was performed based on the different countries. The subjects in 7 studies were divided into domestic and overseas groups. Subgroup meta-analysis showed that in both the domestic or overseas populations, pregnant women with SCH have higher miscarriage risks (RR = 2.00, 95% CI 1.61–2.48 vs. RR = 1.69, 95% CI 1.25–2.29). No heterogeneity was observed in each subgroup (Table 2).

**Table 2:** Subgroup analysis: The prevalence of miscarriage risk in SCH patients without intervention and SCH patients with treatments compared to controls.

	Number of studies	*RR* (95% CI)	*P*	Heterogeneity test
SCH without intervention	7	1.90 (1.59~2.27)	<0.01	*P* = 0.43, *I*^*2*^ = 0%
Domestic	5	2.00 (1.61~2.48)	<0.01	*P* = 0.34, *I*^*2*^ = 12%
Overseas	2	1.69 (1.25~2.29)	<0.01	*P* = 0.36, *I*^*2*^ = 0%
SCH with treatments	4	1.14 (0.82~1.58)	0.43	*P* = 0.61, *I*^*2*^ = 0%
Domestic	2	1.08 (0.76~1.52)	0.67	*P* = 0.66, *I*^*2*^ = 0%
Overseas	2	2.22 (0.75~6.55)	0.15	*P* = 0.89, *I*^*2*^ = 0%

#### (2) Isolated SCH and miscarriage

**Fig 3:**
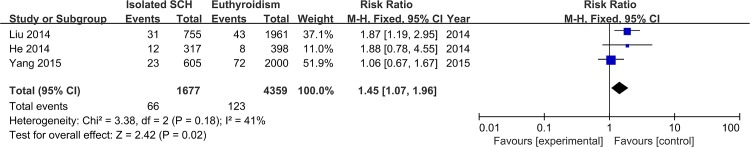
Forest plots for the prevalence of miscarriage risk in isolated SCH patients compared with controls.

Meta-analysis of the 3 studies that reported relevant data on the association between miscarriage and isolated SCH obtained a pooled RR of 1.45 (95%CI 1.07–1.96, P = 0.02), indicating that the prevalence of miscarriage in isolated SCH patients was significantly higher than that in women with normal thyroid function (Fig 3).

#### (3) SCH with treatments and miscarriage

Meta-analysis of 4 studies reported the relevant data on the association between miscarriage and isolated SCH obtained a pooled RR of 1.14 (95%CI 0.82–1.58, P = 0.43), which indicated that there was no statistically significant difference in miscarriage risk of SCH patients accepting effective treatments and pregnant women with euthyroid. Subgroup meta-analysis showed that in both the domestic or overseas populations, SCH with treatments was not associated with miscarriage (RR = 1.08, 95% CI 0.76–1.52, P>0.05 vs. RR = 2.22, 95% CI 0.75–6.55, P>0.05). No heterogeneity was observed in each subgroup with I^2^ = 0% (Table 2).

#### (4) SCH with or without intervention and miscarriage

**Fig 4:**
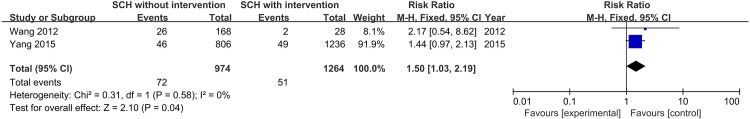
Forest plots of the prevalence of miscarriage risk in SCH without intervention compared to SCH with treatments.

Two studies compared the influence of SCH with treatments and without intervention on the risk of miscarriage. The results of the meta-analysis indicated that compared with patients that underwent effective drug treatments, the prevalence of miscarriage risk in patients without intervention significantly increased with a combined RR = 1.50 (95% CI 1.03–2.19, P = 0.04) (Fig 4).

#### (5) SCH with or without thyroid autoimmunity (TAI) and miscarriage

**Fig 5:**
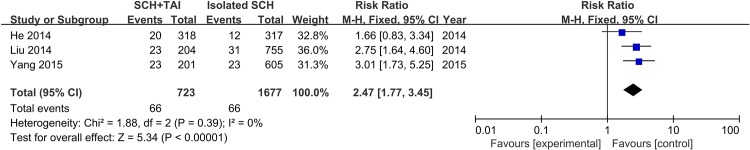
Forest plots for the prevalence of miscarriage risk in SCH with TAI compared to isolated SCH.

Three eligible studies compared the influence of SCH with TAI and isolated SCH on the risk of miscarriage. The result of the meta-analysis indicated that compared with isolated SCH, the prevalence of miscarriage risk in patients with TAI significantly increased with a combined RR = 2.47 (95% CI 1.77–3.45, P<0.01) (Fig 5).

### 3. Sensitivity analysis

**Fig 6:**
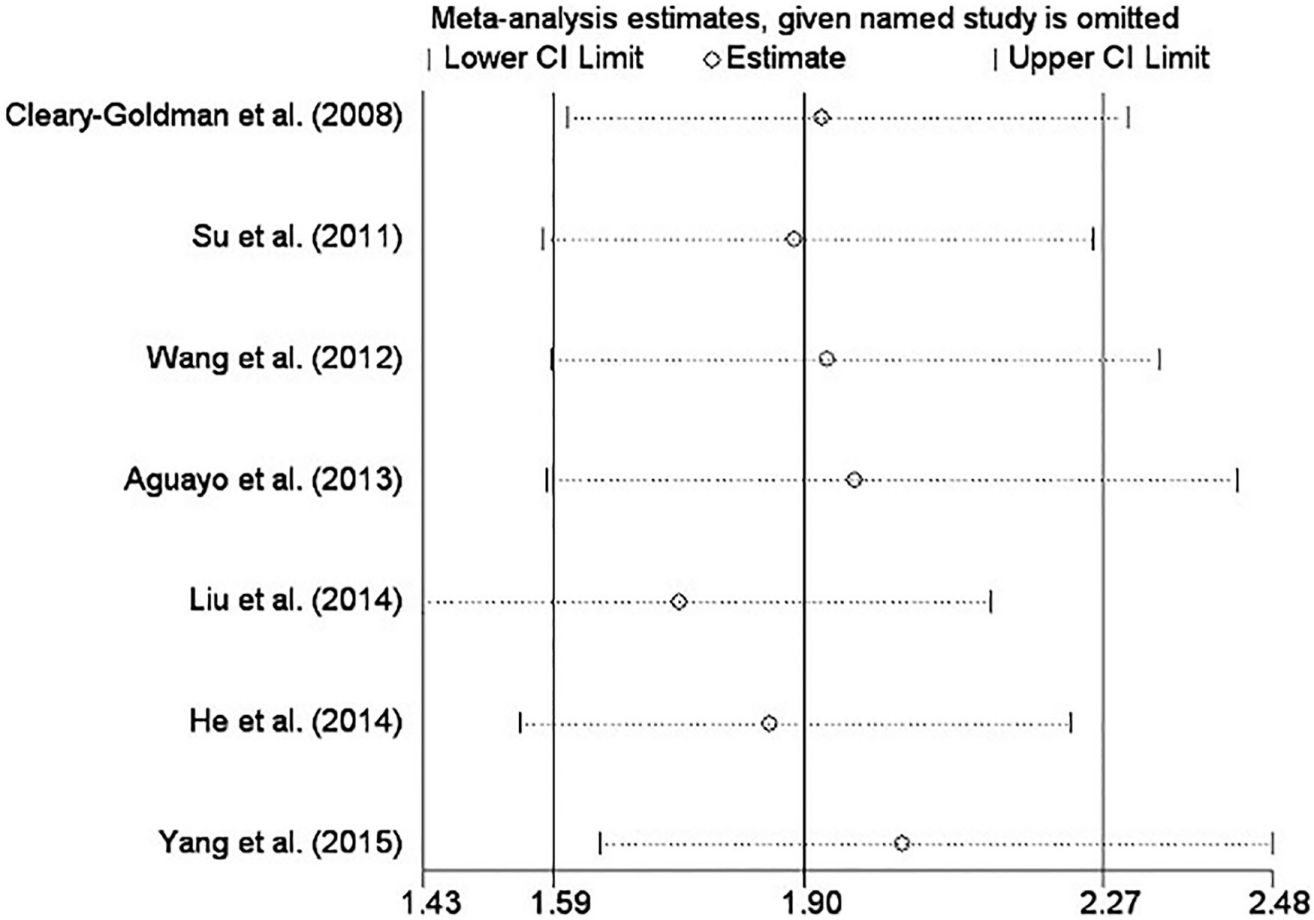
The sensitivity analysis of the included studies reporting relevant data on the association of SCH without intervention and miscarriage.

To assess the effects of each individual research and to verify the stability of the results of the meta-analysis, sensitivity analysis was conducted by removing each study in turn and estimating the overall effect of the remaining studies sequentially. In this meta-analysis, only 7 studies reporting relevant data on the association of SCH without intervention and miscarriage were analysed because the number of studies in the other 4 groups was too few to perform a meaningful sensitivity analysis. After removing each individual study, the pooled RR of the remaining 6 studies were all located in the range of the overall effect, indicating that the results of the meta-analysis showed low sensitivity and high stability (Fig 6).

### 4. Publication bias

**Fig 7:**
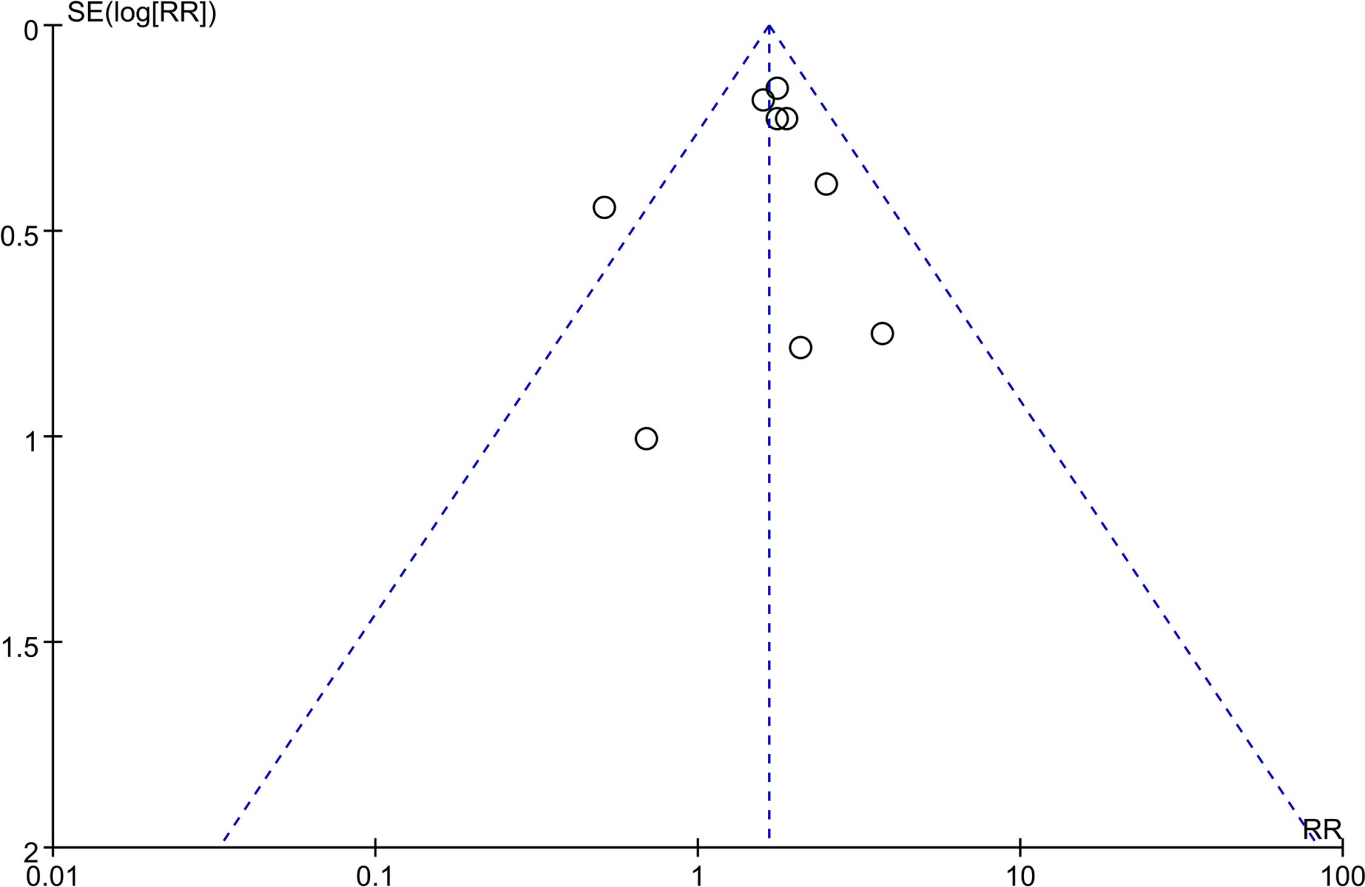
Funnel plots to test the potential publication bias of included studies reporting relevant data on the association of SCH without intervention and miscarriage.

A funnel plot and Begg’s test were performed to assess whether there was a potential publication bias in the included literature. The general symmetry of the funnel plot indicated that there was no obvious publication bias (Fig 7). As the funnel plot is a subjective and straightforward method, we eventually utilized Begg’s test to obtain statistical evidence to verify the absence of publication bias (P = 0.764).

## Discussion

Alterations in the endocrine and metabolic status during pregnancy might contribute to changing levels of thyroid hormones. Several reasons may account for the changes of thyroid function in pregnant women: (1) relative iodine deficiency during pregnancy; (2) the effects of human chorionic gonadotropin (hCG) on the activation of thyroid function stimulates the secretion of thyroid hormones, which could inhibit adenohypophysis function and suppress the levels of thyrotropin; (3) elevated levels of oestrogen during pregnancy, increasing serum thyroid binding globulin (TBG) and raising the concentrations of serum total thyroxine; and (4) the effect of the placenta on thyroxine deiodination[15]. Therefore, the normal reference ranges of TSH for pregnant women differ from those of non-pregnant women. To avoid confusion with euthyroid function or thyroid abnormalities, it is necessary to use specific reference ranges of TSH and thyroid hormones during pregnancy.

Recently, several studies have shown a correlation between SCH in gestation and adverse pregnancy outcomes, including preterm, placental abruption, and gestational diabetes mellitus[8, 16–18]. However, the effect of SCH on the risk of miscarriage remains unclear. In our study, we have systemically reviewed the literature published from 2008–2015, providing a basis for understanding the relationship of gestational SCH with miscarriage risk. We report that compared with euthyroid subjects, women with untreated SCH in early pregnancy show a 1.9-fold risk of miscarriage, suggesting that SCH is useful in predicting miscarriage. However, further studies on this matter may be needed to reveal the mechanisms.

The 2011 Guidelines of the ATA and the 2012 Guidelines of TES recommended that the specific reference ranges for TSH in the early, middle and late stages of pregnancy are 0–2.5 mIU/L, 0.2–3.0 mIU/L, and 0.3–3.0 mIU/L, respectively[14, 19], which has been widely adopted by the international community. However, several recent studies have suggested that it is more reasonable to establish pregnancy-specific diagnostic criteria based on the local TSH levels because the assessment of thyroid function varies widely with region and race[20, 21]. The present analysis included studies using different diagnostic criteria, and compared the results of the ATA standard and specific standard. It has been suggested that with either criteria, once SCH is diagnosed, the risk of miscarriage will increase (OR = 2.04, ATA; 1.58, specific standard). Furthermore, the miscarriage rate based on the ATA standard is higher than that based on the specific standard.

**Fig 8:**
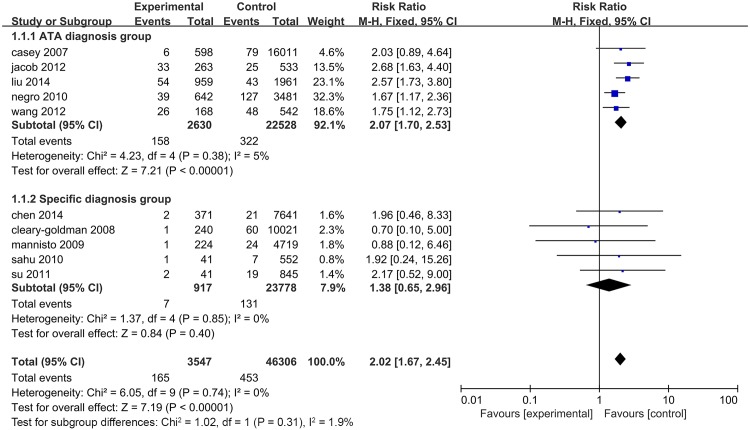
Forest plots for the prevalence of miscarriage risk in untreated SCH patients diagnosed with ATA criteria and specific criteria compared with control(data in forest plots were from the study of Maraka et al.)

Recently, Maraka, et al.[22] reported that pregnancy with SCH is closely associated with a higher foetal mortality (miscarriage rate and stillbirth rate) in a systematic analysis. Data obtained from 3 individual studies based on specific TSH ranges, which included 7 miscarriage cases in 917 SCH patients and 131 cases in 23,778 healthy subjects, showed no significant difference between SCH subjects and healthy controls (RR = 1.38, 95% CI = 0.65–2.96, *P* = 0.40). Each of these studies indicated no significant differences in miscarriage risk between SCH and healthy subjects. In addition, according to the ATA criteria, we conducted a meta-analysis involving 158 cases of miscarriage in 2,630 individuals with SCH and 322 cases in 22,528 healthy individuals. This suggested an elevated risk of miscarriage for SCH pregnant women compared with healthy controls (RR = 2.07, 95% CI 1.70–2.53, *P*<0.01) (Fig 8). These results suggested that miscarriage might be undetected due to the use of specific SCH criteria. Therefore, it is more beneficial to pregnancy if the levels of TSH are controlled to under 2.5 mIU/L in early pregnancy.

Currently, it is controversial whether pregnancy with SCH should be treated. ATA Guidelines recommended that TPOAb-positive subjects with TSH>2.5 mIU/L and normal FT_4_ or subjects with TSH>10.0 mIU/L should be treated with L-thyroxine (L-T_4_)[14]. For TPOAb-negative subjects with TSH>2.5 mIU/L, there is not enough evidence to support the treatment of L-T_4_. The TES guidelines recommended that subjects with TSH>2.5 mIU/L and normal FT_4_, regardless of TPOAb positivity, should accept L-T_4_ treatment[1]. The present study suggested that pregnant women with SCH who underwent L-T_4_ treatment had a significantly lower miscarriage rate than SCH subjects without treatment, whereas no significant difference was observed between patients with treatment and healthy individuals. This indicates that the treatment rate of SCH during pregnancy should be improved, as it helps to avoid miscarriage. Meanwhile, the miscarriage risk is significantly higher in pregnant women with SCH but who are not TPOAb-positive, which suggests that we also cannot ignore the management of isolated SCH patients.

Thyroid autoantibodies are important indexes to evaluate the status of thyroid autoimmunity. TPOAb plays a role in thyroid damage by activating complement-dependent cytotoxicity (CDC), antibody-dependent cell-mediated cytotoxicity (ADCC), and the killing effect of T-cells. Autoimmune thyroiditis is considered the major cause of SCH. Glinoer et al.[23] showed that thyroid autoantibodies indicate the abnormality of immune function, which induces miscarriage by unstable placenta implantation. Negro et al.[24] suggested that thyroid autoantibodies imply poor thyroid function during pregnancy, which might result in clinical and subclinical hypothyroidism, increasing the risk of miscarriage. Specifically, SCH subjects with TPOAb positivity had a significantly higher miscarriage risk than isolated SCH subjects. Therefore, more attention is required in these populations, and the L-T4 should be considered early to prevent poor pregnancy outcomes.

Our study has several limitations. Firstly, given language limitations, more Chinese studies were included, leading to a certain selection bias. Secondly, several studies used the fetal loss rather than miscarriage as a pregnancy outcome, which were excluded as a result of failure to extract data. Additionally, the SCH diagnostic criteria of the included studies varied with periods and regions. Although we performed subgroup analyses based on the different criteria, the effects cannot be eliminated completely. What is more, among 4 studies concerned about the treatments, 3 of them set up the targets according to ATA recommended criteria (TSH<2.5 mIU/L at the first trimester and TSH<3.0 mIU/L at the second and third trimesters), while one research set up the target according to the pregnancy-specific criteria based on the local TSH levels. Given the limitations above, more large-scale prospective surveys are needed.

## Conclusion

SCH is a risk factor for miscarriage in women before 20 weeks of pregnancy, and early treatments can reduce the miscarriage rate. With either diagnostic criterion, the risk of miscarriage will increase once SCH is diagnosed. However, specific diagnostic criteria might result in increased diagnosis of miscarriage that would have been missed otherwise. The miscarriage rate is higher in isolated SCH subjects and increases further in SCH pregnant women with positive thyroid autoantibodies.

## Supporting information

S1 File PRISMA 2009 checklist. (DOC)
